# Cognition and Subjective Age Predict Physical Activity Engagement: A Longitudinal Study of Impaired Older Adults

**DOI:** 10.1093/geroni/igab046.2883

**Published:** 2021-12-17

**Authors:** Claire Growney, Xianghe Zhu, Shevaun Neupert

**Affiliations:** 1 Washington University in St. Louis, Saint Louis, Missouri, United States; 2 North Carolina State University, North Carolina State University, North Carolina, United States; 3 North Carolina State University, Raleigh, North Carolina, United States

## Abstract

Physical activity is an important factor in preventing or slowing cognitive decline. However, the predictors of fluctuations in physical activity in a population that is already experiencing cognitive impairment is not well understood. Subjective age, such as how old one feels, has been tied to many health indicators in cognitively intact populations. Thus, we focused on the within-person associations between subjective age and physical activity as they unfold over time within a sample of cognitively impaired participants. The current study examined 400 reports from measurement burst data consisting of 5 weekly surveys conducted twice across 6 months from 68 cognitively impaired participants (M age = 70.14 (6.63), range = 60-92). Participants completed a battery of cognitive tests at baseline. At each weekly assessment, participants reported on their physical activity (e.g., exercise, outdoor, flexibility, and strength activities; Yes/No) and subjective age with respect to how old they feel overall and how old they feel mentally. There were longitudinal decreases in physical activity across the bursts, but on occasions when participants felt younger overall or younger mentally there were increases in physical activity. In addition, the effects of mental subjective age depended on cognitive ability, with those scoring lower in cognitive ability appearing to benefit the most from decreases (feeling younger) in mental subjective age. These findings suggest that perceptions of aging, especially within the domain of mental age, are tied to physical activity engagement for older adults with cognitive impairment.

